# The Southern Dental Association

**Published:** 1886-08

**Authors:** 


### Editorial, Etc.

THE SOUTHERN DENTAL ASSOCIATION.—The eighteenth annual meeting of this Association was held in Nashville, Tennessee, July 28th, 29th and 30th, 1886, and was not only largely attended, but was one of the most interesting sessions ever held. The president, Dr. Wardlaw, of Augusta, Ga., in his address referred as follows to associative effort:

"I wish here to say, that in my estimation, no one instrumentality exerts more potent influence in promoting the dignity of our profession than these association gatherings.

Associative effort seems to be the characteristic feature of this day of progress. Everywhere and on all sides we have associations and conventions—agricultural, commercial, scientific, literary and religious.

Man is a gregarious animal. No one lives to himself. We cannot stand alone. We are mutually dependent. Independence is a myth. Even our every word is learned from, and belongs to, some one else.

Originality is a scarce article. All of our ideas are evolved from those of others. We may pride ourselves upon the conceptions of our fertile brains, but a strict analysis of our mental processes would reveal to us the thoughts of other minds disguised in the habiliments of our own words. There must be a reciprocity of ideas, methods and experiences and here we have it.

As natural fruits of our association, I mention laws regulating the practice of dentistry, State examining boards, the national association of dental examining boards and the national association of faculties of dental colleges, all exercising a reciprocal influence upon education, that foundation stone upon which our professional edifice should stand. The laws gave us our examining and licensing boards. As their usefulness increased, and their functions enlarged, the State boards realized the importance of co-operation and correspondence of action, and hence was evolved the "Association of Examining Boards." These boards, National and State, exerted such marked influence upon the colleges, as to cause them to advance their standards and leagues together in their "Association of Faculties."

This is the direction in which this good work should go on. Let our boards year by year, systemize their plans and perfect their workings until they are in entire accord and harmony with each other, and until a license issued in one State will be of binding force in all the States and the diplomas of all the colleges will be received with respect and authority wherever presented.

Having reached this point we will be prepared to take the grand culminating step which will place us clearly in the lead of the medical profession. I mean, and this is the point, if point it has, of my address, that the dental profession should confer upon its worthy students a national degree which should subordinate or abolish the various degrees of D. D. S., D. M. D., L. D. D., etc., and be analagous in rank, reputation and dignity to the great English degree, "Fellow of the Royal College of Physicians." This is the great desideratum to which we must direct our efforts, but of which I havn't time to say more now. It may be unknown to some here, that we are even now, leading the medical fraternity in matters of law and education. Almost nothing is being done by it in these directions."

The editor of this JOURNAL received the following very complimentary notice in the *Daily American*, of Nashville, report of the proceedings:

"All of the meetings of the body will be open to the general public, and all physicians and dentists in the city will meet with a hearty welcome. There will be present a number of men who have distinguished themselves and added lustre to their profession. One of these is Dr. F. J. S. Gorgas, Dean of the Dental Department of the University of Maryland. He is one of the oldest dentists in America and is the honored author of many well-known text books."

A communication from the National Association of Dentists inviting a reciprocal committee to confer for future closer relations between the two bodies was with one voice firmly, but respectfully rejected.

The following officers were elected for the ensuing year, the next meeting to be held at Old Point Comfort, Va.

W. W. H. Thackston, of Farmville, Va., President; B. H. Catching, of Atlanta, Ga., first vice-President, Rollo Knapp, of New Orleans, La., second vice-President; W. H. Richards, of Chattanooga, Tenn., third vice-President; J. Y. Crawford, of Nashville, Tenn., Corresponding Secretary; Louis P. Dotterer, of South Carolina, Secretary; H. R. Lawrence, of Athens, Ga., General Treasurer. Executive Committee—J. B. Woodley, of Norfolk, Va., J. H. Moore, of Richmond, Va , and E. S. Chisholm, of Alabama.

